# Immunoregulatory effects of testosterone supplementation combined with exercise training in men with Inclusion Body Myositis: a double‐blind, placebo‐controlled, cross‐over trial

**DOI:** 10.1002/cti2.1416

**Published:** 2022-09-22

**Authors:** Jerome D Coudert, Nataliya Slater, Anuradha Sooda, Kelly Beer, Ee Mun Lim, Conchita Boyder, Rui Zhang, Frank L Mastaglia, Yvonne C Learmonth, Timothy J Fairchild, Bu B Yeap, Merrilee Needham

**Affiliations:** ^1^ Centre for Molecular Medicine and Innovative Therapeutics Murdoch University Murdoch WA Australia; ^2^ Perron Institute for Neurological and Translational Science Nedlands WA Australia; ^3^ School of Medicine University of Notre Dame Fremantle WA Australia; ^4^ Department of Clinical Biochemistry, Pharmacology and Toxicology, PathWest Laboratory Medicine QEII Medical Centre Nedlands WA Australia; ^5^ Discipline of Exercise Science Murdoch University Murdoch WA Australia; ^6^ Medical School University of Western Australia Perth WA Australia; ^7^ Department of Endocrinology and Diabetes Fiona Stanley Hospital Perth WA Australia; ^8^ Department of Neurology Fiona Stanley Hospital Perth WA Australia

**Keywords:** autoimmunity, clinical trial, exercise therapy, Inclusion Body Myositis, testosterone

## Abstract

**Objectives:**

Sporadic Inclusion Body Myositis (IBM) is an inflammatory muscle disease affecting individuals over the age of 45, leading to progressive muscle wasting, disability and loss of independence. Histologically, IBM is characterised by immune changes including myofibres expressing major histocompatibility complex molecules and invaded by CD8^+^ T cells and macrophages, and by degenerative changes including protein aggregates organised in inclusion bodies, rimmed vacuoles and mitochondrial abnormalities. There is currently no cure, and regular exercise is currently the only recognised treatment effective at limiting muscle weakening, atrophy and loss of function. Testosterone exerts anti‐inflammatory effects, inhibiting effector T‐cell differentiation and pro‐inflammatory cytokine production.

**Methods:**

We conducted a double‐blind, placebo‐controlled, cross‐over trial in men with IBM, to assess whether a personalised progressive exercise training combined with application of testosterone, reduced the inflammatory immune response associated with this disease over and above exercise alone. To assess intervention efficacy, we immunophenotyped blood immune cells by flow cytometry, and measured serum cytokines and chemokines by Luminex immunoassay.

**Results:**

Testosterone supplementation resulted in modest yet significant count reduction in the classical monocyte subset as well as eosinophils. Testosterone‐independent immunoregulatory effects attributed to exercise included altered proportions of some monocyte, T‐ and B‐cell subsets, and reduced IL‐12, IL‐17, TNF‐α, MIP‐1β and sICAM‐1 in spite of interindividual variability.

**Conclusion:**

Overall, our findings indicate anti‐inflammatory effects of exercise training in IBM patients, whilst concomitant testosterone supplementation provides some additional changes. Further studies combining testosterone and exercise would be worthwhile in larger cohorts and longer testosterone administration periods.

## Graphical abstract

This is a double‐blind, placebo‐controlled, cross‐over trial in 14 men with IBM, to assess whether a personalised progressive exercise training combined with topical application of testosterone, reduced the inflammatory immune response associated with this disease over and above exercise alone. Testosterone supplementation resulted in significant reduction in the classical monocyte subset as well as eosinophils, and of MIP‐1β. Testosterone‐independent immunoregulatory effects attributed to exercise included altered proportions of monocyte, T and B cell subsets, and reduced concentrations of pro‐inflammatory IL‐12, IL‐17, TNF‐α and sICAM‐1.

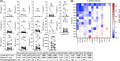

## Introduction

Sporadic Inclusion Body Myositis (IBM) is the most common acquired skeletal muscle disease associated with ageing, occurring more commonly in men.^1^ The progressive disease course results in muscle loss and debilitating loss of mobility, negatively impacting quality of life.^2^ Based on evidence provided by previous studies in IBM, it is broadly recognised in the field that regular personalised exercise programme is currently the most effective intervention available to preserve and prolong muscle functional capacity.^3^ Exercise training has been shown to be safe for IBM patients^4^, ^5^, ^6^; it is the only treatment recognised to impact disease progression. Histologically, the disease is characterised by a mononuclear cell infiltrate in the endomysium and invading non‐necrotic muscle fibres, up‐regulated MHC class I and II expression on the sarcolemma as well as within the sarcoplasm, and elevated presence of pro‐inflammatory cytokines and chemokines. These autoimmune manifestations are combined with degenerative changes in muscle fibres comprising autophagic rimmed vacuoles, multiprotein aggregates forming inclusion bodies and mitochondrial abnormalities. The invading lymphocytes are predominantly cytotoxic CD8^+^ T cells, and also composed of CD4^+^ T cells, B cells and dendritic cells (DC).^2^, ^7^, ^8^ The presence of autoantibodies directed against cytosolic 5′‐nucleotidase 1A (NT5C1A) has been reported in 33–76% of IBM patients.^9^ NT5C1A is involved in the conversion of adenosine monophosphate (AMP) to adenosine and catalyses the hydrolysis of nucleotides to nucleosides; it is most abundant in skeletal muscles and is aberrantly distributed in rimmed vacuoles and degenerative areas in IBM muscles.^10^ The pro‐inflammatory and pathogenic role of anti‐NT5C1A in IBM remains unclear.

Multiple lineages of immune cells possess receptors for sex hormones that regulate their function.^11^ It is also well documented that sexual dimorphism exists with multiple autoimmune conditions, usually with a higher prevalence in women.^11^ However, a strong correlation has been observed between hypogonadism in men and an increased incidence of autoimmunity,^12^ suggesting that androgens may have an immunomodulatory function. Testosterone elicits an anti‐inflammatory effect, suppressing Th17 and Th1 responses, whilst its effect on Th2 immunity remains ambiguous.^13^

Cytokines and chemokines are soluble mediators that control immune responses; they are produced both by immune and by nonimmune cells.^14^ IBM patients exhibit elevated blood concentration of multiple Th1‐type cytokines and chemokines.^15^ Th1 cells, which are involved in cellular immune responses against intracellular pathogens, are abundant in inflammatory autoimmune conditions. The presence of Th17 cells has been implicated in the pathogenesis of multiple autoimmune disorders; the secretion of IL‐17 by this CD4^+^ T‐cell subset leads to macrophage and neutrophil attraction within the affected tissues, which further reinforces the inflammatory response.^16^ However, Th17 cells are not increased in IBM patients' blood, and only small numbers have been detected within muscle infiltrates.^15^, ^17^ Testosterone has been shown to reduce cytokines responsible for systemic inflammation such as tumor necrosis factor‐α (TNF‐α), interleukin‐1β (IL‐1β) and IL‐6, and conversely, to promote the production of immunosuppressive IL‐10.^18^, ^19^ Therefore, these anti‐inflammatory effects suggest that testosterone may play a beneficial role in chronic inflammatory condition such as IBM.

In male adults, the production of testosterone decreases with age, with average concentrations in the elderly people corresponding to the lower end of the range observed in middle‐aged men.^20^ This decrease is in part due to the higher prevalence of obesity and medical comorbidities with ageing.^21^, ^22^ A longitudinal study found that approximately 20% of men over the age of 60 and 50% over the age of 80 display hypogonadism, associated with lower skeletal muscle mass and strength.^23^ A cross‐sectional study of 59 men with different forms of muscular dystrophy or myopathy, including 17 with IBM, found that half of the men had total testosterone concentrations below the lower limit of the reference range.^24^ Previous studies conducted in healthy young and middle‐aged men showed that frequent exercise training combined with testosterone supplementation improved skeletal muscle mass, strength and performance to a greater extent than exercise alone.^25^, ^26^ A similar additive effect of testosterone and exercise was reported in older men with increased muscle performance, despite the absence of significant lean body mass gain.^27^, ^28^ A pilot study investigating the effect of a synthetic analogue of testosterone, oxandrolone, in IBM patients reported a near‐statistically significant, whole body strength improvement.^29^ Whilst regular exercise is a recognised intervention that effectively reduces the loss of muscle mass and function in IBM patients,^3^, ^30^ the impact of combined testosterone supplementation and exercise has not been investigated in IBM.

Considering the anti‐inflammatory actions of testosterone and its additive impact on performance improvement for muscles undergoing exercise training, we hypothesised that combining testosterone supplementation with exercise training would be more beneficial for men with IBM than exercise alone, in reducing the inflammatory immune response. We therefore designed a 26‐week double‐blind, cross‐over randomised controlled trial (RCT) to which we enrolled 14 men with a diagnosis of IBM. Participants were required to undertake a personalised progressive exercise training routine for the entire duration of the study, whilst receiving either testosterone or placebo for 12 weeks each, with a 2‐week washout between study arms.^31^ To quantify the level of inflammation and investigate immune changes related to treatment, we measured the size of immune cell populations and the concentration of pro‐inflammatory cytokines and chemokines in the blood at baseline and after each treatment period.

## Results

### Patient demographics, randomisation and effect of testosterone supplementation on circulating androgen concentration

The age of the 14 participants at baseline ranged between 45 and 81 years (65 ± 14 years [mean ± SD]), with duration of IBM symptoms ranging from 4 to 23 years (11 ± 7 years [mean ± SD]; Supplementary table 1). The testosterone supplementation period resulted in increased concentrations of both testosterone (median: + 9.5 nm) and DHT (median: + 3.2 nm), whilst only minor changes were measured following administration of the placebo (Supplementary figure 3a). The calculated Spearman's rank correlation coefficients did not provide evidence of correlation of testosterone or DHT variation with age (Supplementary figure 3b); however, the variation of testosterone concentration, but not of DHT, was inversely correlated with disease duration (*r* = −0.575; *P* = 0.03; Supplementary figure 3c). These results suggest that the testosterone supplementation has a less potent effect on testosterone blood content elevation as disease duration, but not with patient's age, increases.

### Effect of testosterone supplementation on immune cell population size and differentiation in the blood

**Figure 1:**
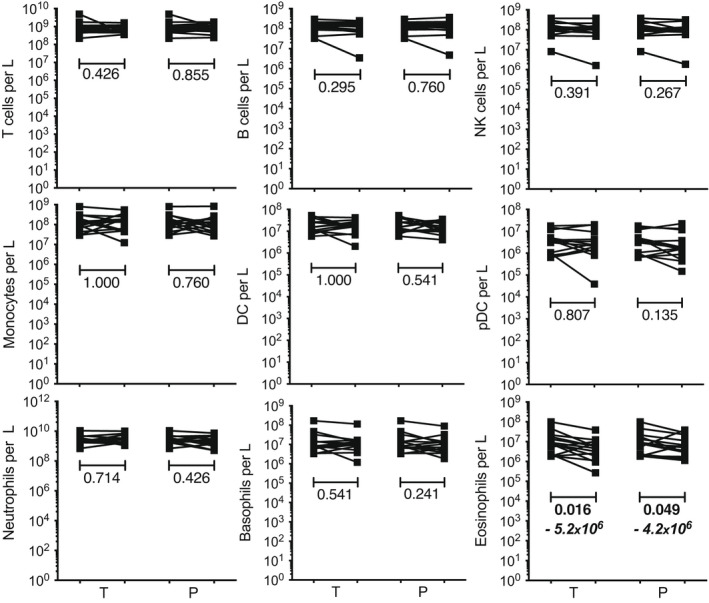
Effect of testosterone supplementation on cell numbers within leukocyte populations. The number of cells in leukocyte populations, expressed per litre of blood, were measured at baseline and after each study arm (T, testosterone; P, Placebo). Each line shows the count variation measured for a participant during the indicated study arm. The statistical significance of the changes was assessed using the Wilcoxon matched‐paired signed rank test; where changes were found to be significant (*P* < 0.05), the *P*‐values are highlighted in bold and the median count variations are indicated.

By the end of the testosterone arm, none of the major populations of leukocytes had significantly changed cell counts, with the exception of eosinophils which decreased by 5.2 x 10^6^ cells L^−1^ of blood (*P* = 0.016; Figure 1). A marginally significant and more limited reduction (−4.2 x 10^6^ cells L^−1^, *P* = 0.049) was also observed after application of the placebo, suggesting that this variation may have occurred independently of testosterone.

**Figure 2:**
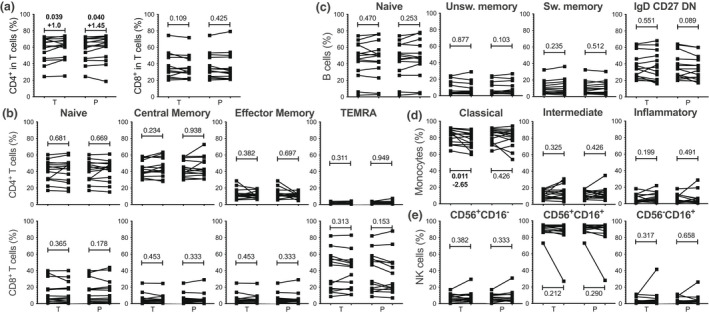
Effect of testosterone supplementation on leukocyte subset proportions. The proportion of immune cell subsets was calculated at baseline and after each study arm (T: testosterone, P: Placebo). Each line shows the variation of frequency of the indicated subset measured for a participant during the indicated study arm. **(a)** Proportion of CD4^+^ and of CD8^+^ T cells within total T cells. **(b)** Proportion of naïve, central memory, effector memory and TEMRA cells measured within CD4^+^ T cells (top panels) and CD8^+^ T cells (bottom panels). **(c)** Proportion of naïve, unswitched (Unsw.) memory, switched (Sw.) memory and IgD, CD27 DN cells within B cells. **(d)** Proportion of the classical, intermediate and inflammatory subsets within monocytes. **(e)** Proportion of NK cell subsets based on differential CD56 and CD16 expression. The statistical significance of the changes was assessed using the Wilcoxon matched‐paired signed rank test; where changes were found to be significant (*P* < 0.05), *P*‐values are highlighted in bold and the median percentage variations are indicated.

We assessed whether discrete changes hidden at the scale of the leukocyte populations could be detected within specific subsets. The proportion of the CD4^+^ subset amongst T cells increased slightly after the testosterone supplementation period (+1%), but this effect was not testosterone‐dependent as a 1.45% increase was also detected following the placebo arm (Figure 2a). The stages of CD4^+^ and CD8^+^ T‐cell differentiation can be discriminated based on the differential expression of CCR7 and CD45RA; naïve cells are CCR7^+^CD45RA^+^ whilst memory subsets are partitioned into central memory (CCR7^+^CD45RA^−^), effector memory (CCR7^−^CD45RA^−^) and late‐differentiated TEMRA (CCR7^−^CD45RA^+^). We did not detect significant alterations of the subset repartition between these different stages of differentiation in either CD4^+^ or CD8^+^ T cells after the testosterone supplementation period (Figure 2b).

Within B cells, the naïve and memory subsets harbour different expression patterns of IgD and CD27.^32^ Naïve B cells are CD27^−^IgD^high^ whilst memory cells are CD27^+^IgD^+^ or CD27^+^IgD^−^ depending on their unswitched or switched state, respectively. A third memory subset of late‐differentiated cells lacks both IgD and CD27 (IgD, CD27 double‐negative [DN]). Although some changes were noted in some of the participants, none of the B‐cell subset proportions were significantly altered overall following testosterone supplementation (Figure 2c).

In monocytes, the expression patterns of CD14 and CD16 discriminate the classical (CD14^high^CD16^−^), the intermediate (CD14^high^CD16^+^) and the nonclassical (or inflammatory, CD14^low^CD16^+^) subsets. A significant reduction in the proportion of the classical monocyte subset was observed following the testosterone arm (−2.65%, *P* = 0.011), but not the placebo arm (Figure 2d). This decrease was modest considering that this monocyte subset accounted for an average of 82% of total monocytes (median 84.75%, Supplementary table 2) at baseline. However, the intermediate and nonclassical subsets were not significantly altered.

Natural killer (NK) cells can be divided into CD56^+^CD16^+^, CD56^high^CD16^−^ and CD56^low^CD16^high^ subsets that possess distinct inflammatory and cytotoxic capabilities. In spite of variations observed in some participants, we did not find significant changes in NK cell subset proportions following testosterone supplementation (Figure 2e).

### Effect of testosterone supplementation on blood concentrations of pro‐inflammatory cytokines and chemokines

**Figure 3:**
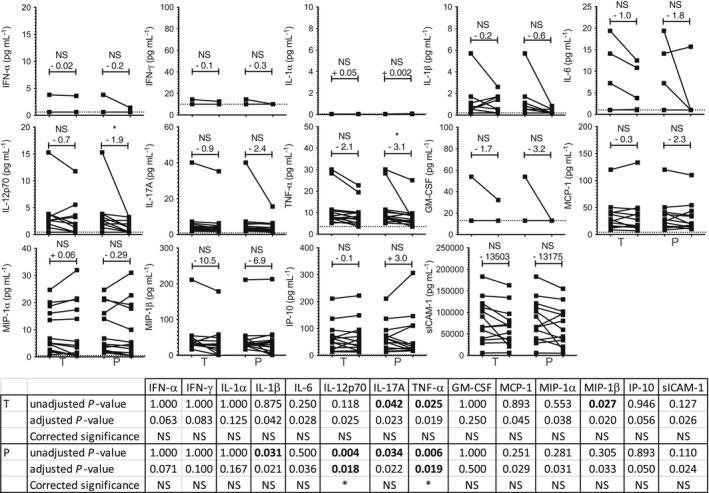
Effect of testosterone supplementation on pro‐inflammatory cytokines and chemokines. Graphs: The concentrations of the indicated cytokines and chemokines were measured at baseline and after each study arm (T, testosterone, P, placebo). Each line shows the variation of concentration of the indicated molecule measured for a participant during each study arm. Above each condition is indicated the variation mean (in pg mL^−1^) and the statistical significance (*: *P* < 0.05; NS: not significant). Table: the statistical significance of the changes assessed using the Wilcoxon matched‐paired signed rank test are indicated; *P*‐values <0.05 are highlighted in bold. To account for multiple comparison analysis, *P*‐values were adjusted using the Holm–Bonferroni's correction method and the corrected statistical significance result are indicated (*, significant when unadjusted *P*‐value < adjusted *P*‐value; NS, not significant).

Following testosterone supplementation, we found a trend for MIP‐1β reduction (mean, −10.5 pg mL^−1^, unadjusted *P* = 0.027) although this change was found to be not significant after correction for multiple comparison analysis was applied. Other changes detected with unadjusted, but not corrected *P*‐values, that affected IL‐17A and TNF‐α were detected after both the testosterone and placebo arms, whilst IL‐1β and IL‐12p70 were reduced following the placebo but not after the testosterone arm (Figure 3), and therefore were not androgen‐dependent.

### Correlation of immune changes with testosterone concentration in blood

Considering the high interindividual variability in blood testosterone concentration and in immune changes in response to supplementation, we investigated whether we could identify correlations between these variables. Eosinophils ranked as the leukocyte population with the strongest, albeit moderate, Spearman's correlation *r value* (*r* = −0.2825) with testosterone concentration, although without reaching the significance threshold (*P* = 0.081). TEMRA and effector memory CD4^+^ T cells were the two only leukocyte subsets exhibiting significant negative correlation with testosterone (*r* = −0.5043, *P* = 0.001 and *r* = −0.3529, *P* = 0.028, respectively). With regard to the soluble immune mediators, we found that the concentrations of TNF‐α (*r* = −0.4096, *P* = 0.009) and MCP‐1 (*r* = −0.3868, *P* = 0.015) were inversely correlated with testosterone (Supplementary figure 4).

Considering that some of the immunological changes detected were not restricted to the testosterone arm, and that the identified correlation between testosterone concentration and immunological parameters did not translate into the corresponding immune changes following the blood testosterone increase that resulted from supplementation, we proposed that the exercise programme rather than testosterone supplementation had induced these anti‐inflammatory effects.

### Analysis of the changes in immune cell populations following the 26‐week exercise training

**Figure 4:**
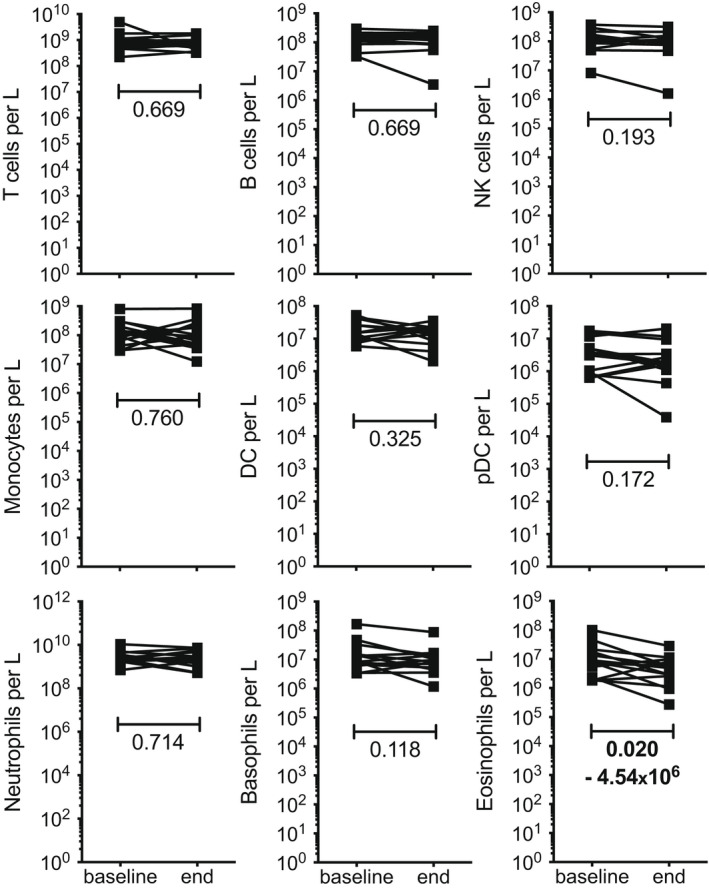
Variation of cell numbers within leukocyte populations during the course of the study. The cell numbers in each leukocyte population, expressed per litre of blood, were measured at baseline and endpoint of the study. Each line shows the count variation measured for a participant during the study. The statistical significance of the changes was assessed using the Wilcoxon matched‐paired signed rank test; where a change was found to be significant (*P* < 0.05), the *P*‐values are highlighted in bold and the median count variation value is indicated.

We compared the immune cell population count at study baseline and endpoint. Again, we found a reduction in eosinophils (median: −4.54 x 10^6^ L^−1^), with decreased numbers observed in 11 of the 14 participants (Figure 4 and Supplementary table 2). Some participants displayed changes in other immune cell populations, but not at a significant level overall.

**Figure 5:**
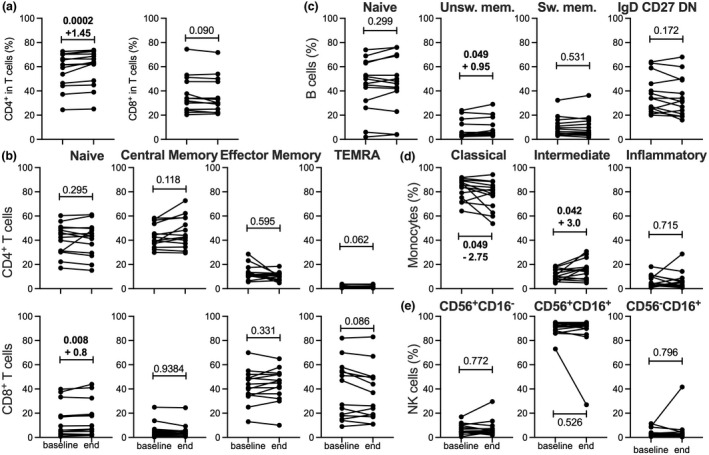
Variation of leukocyte subsets during the course of the study. The proportion of immune cell subsets was calculated at baseline and endpoint of the study. Each line pairs the frequencies of the indicated subset measured for a participant. **(a)** Proportion of CD4^+^ and of CD8^+^ cells within total T cells. **(b)** Proportion of naïve, central memory, effector memory and TEMRA cells measured within CD4^+^ T cells (top panels) and CD8^+^ T cells (bottom panels). **(c)**: Proportion of naïve, unswitched memory, switched memory and IgD, CD27 DN cells within B cells. **(d)** Proportion of the classical, intermediate and inflammatory subsets within monocytes. **(e)** Proportion of CD56^+^CD16^−^, CD56^+^CD16^+^ and CD56^−^CD16^−^ subsets within NK cells. The statistical significance of the changes was assessed using the Wilcoxon matched‐paired signed rank test; where changes were found to be significant (*P* < 0.05), *P*‐values are highlighted in bold and the median variations are indicated.

We assessed whether additional changes could be identified in restricted leukocyte subsets. The frequency of the CD4^+^ subset amongst T cells increased, with a median variation of +1.45% (Figure 5a and Supplementary table 2) but this was not associated with a significant decrease of the CD8^+^ T‐cell subset due to interindividual variability. None of the naïve/memory subsets were affected in CD4^+^ T cells, whilst in CD8^+^ T cells, naïve cells slightly increased (median: +0.8%; Figure 5b). In B cells, the proportion of unswitched memory cells slightly increased (median: +0.95%), whereas the naïve and the other memory subsets remained unchanged (Figure 5c).

Analysis of the monocytes revealed a decreased proportion of the classical subset (median: −2.75%) associated with a higher proportion of the intermediate subset (median: +3.0%; Figure 5d). No changes within the NK cell subsets were observed (Figure 5e).

Overall, these results indicate that the personalised exercise training programme was associated with small‐to‐moderate immune cell changes, including a reduced eosinophil population and altered differentiation of a limited number of subsets within monocytes, T cells and B cells.

### Assessment of changes in autoantibodies during the study

We found that 10 of the 14 participants were seropositive for anti‐C5N1A antibodies. Analysis of the antibody isotypes indicated that 10 participants had IgG, 4 had IgA and 8 possessed IgM. Apart from individual variations observed in a few participants, the concentration of anti‐C5N1A autoantibodies was not significantly altered overall (Supplementary figure 5).

### Analysis of serum concentration of pro‐inflammatory cytokines and chemokines following the 26‐week exercise period

**Figure 6:**
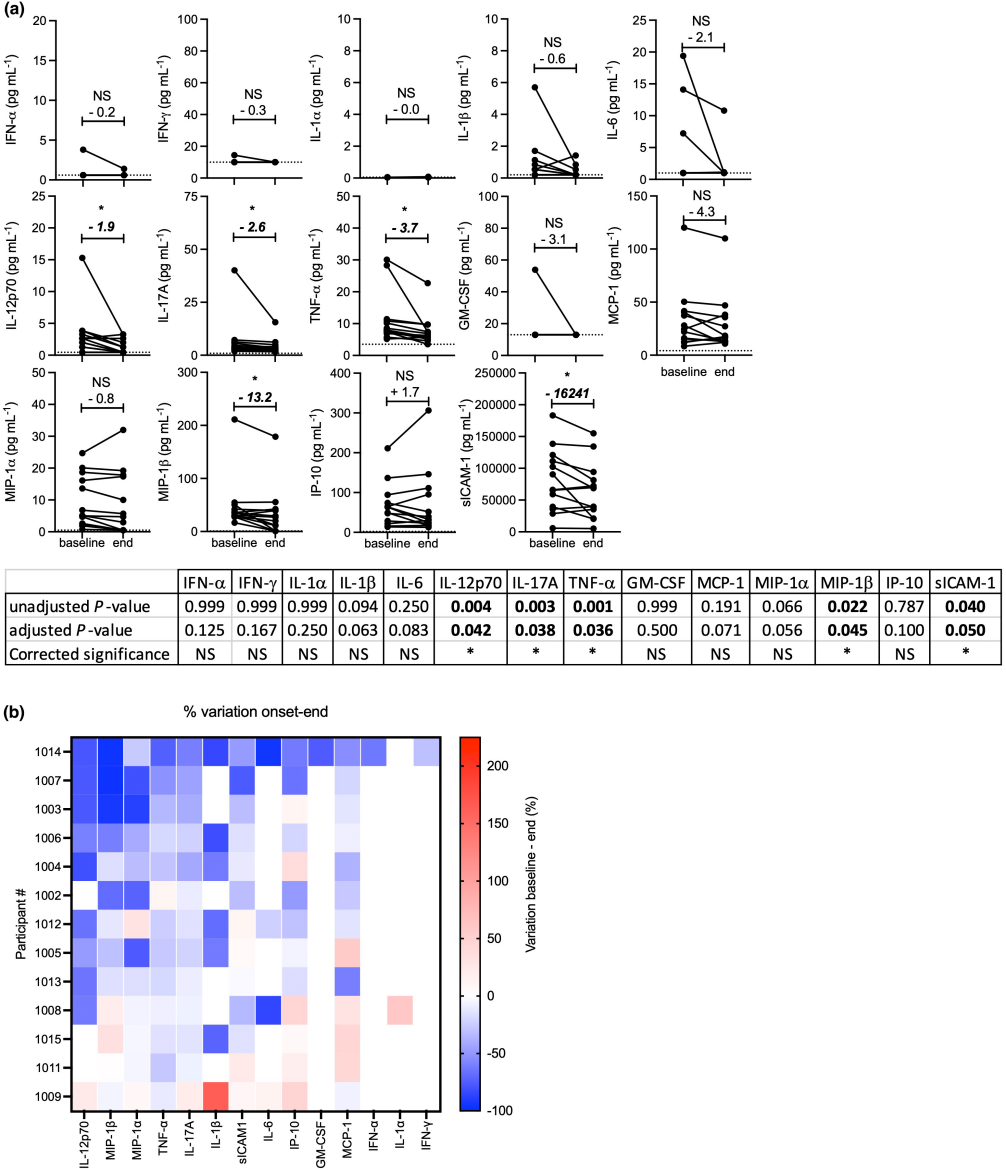
Variation of pro‐inflammatory cytokines and chemokines concentrations during the course of the study. The concentrations of cytokines and chemokines were measured at baseline and endpoint of the study. **(a)** Graph: The lines pair the concentrations measured for each participant. The mean variation of concentration (in pg mL^−1^) is indicated above each dataset. Table: the statistical significance of the changes assessed using the Wilcoxon matched‐paired signed rank test are indicated; *P*‐values < 0.05 are highlighted in bold. To account for multiple comparison analysis, *P*‐values were adjusted using the Holm‐Bonferroni's correction method and the corrected statistical significance result are indicated (*: significant when unadjusted *P*‐value < adjusted *P*‐value; NS: not significant). **(b)** Heatmap representation of participants' individual changes in the concentration of pro‐inflammatory cytokines and chemokines during the study. The changes for each of these molecules are expressed as % of variation calculated as follows: [(concentration at endpoint – concentration at baseline)/ (concentration at baseline)] x 100. The cytokines and chemokines were ranked according to their change in concentration across all the participants, from the most to the least decreased (*x*‐axis, left to right, respectively). The participants were ranked according to the combined anti‐inflammatory effect observed across all the cytokines and chemokines, from the strongest to the weakest combined reduction (*y*‐axis, top to bottom, respectively).

The trend for IL‐17A, TNF‐α, and MIP‐1β reduction measured during the testosterone supplementation arm became significant after the whole course of the study (mean: −2.6, −3.7 and −13.2 pg mL^−1^, respectively). In addition, we found reduced concentrations of IL‐12p70 (mean: −1.9 pg mL^−1^) and sICAM‐1 (median: −16 241 pg mL^−1^; Figure 6a). The reduction of IL‐1β observed during the placebo arm was not significant over the whole duration of the study (*P* = 0.09) due to interindividual variability.

We investigated the interparticipant response variability using a heatmap representation where participants were ranked from higher to lower responder to the exercise training in term of reduced inflammation. We could identify at the upper end participants who demonstrated decreased concentration of most of the pro‐inflammatory cytokines and chemokines tested, whereas at the lower end, participants displayed increased concentrations of several of these molecules (Figure 6b). There was no association of these individual patterns of response with age, disease duration or testosterone/placebo randomisation sequence (data not shown). Also, we ranked the cytokines and chemokines according to the consistency of the reduction in their concentration across all the participants over the course of the study. During the study, the pro‐inflammatory molecule showing the most consistent relative reduction was IL‐12p70 then MIP‐1β, MIP‐1α, TNF‐α, IL‐17A, IL‐1β and sICAM‐1 (Figure 6b).

### Correlation of immune changes with blood creatine kinase

**Figure 7:**
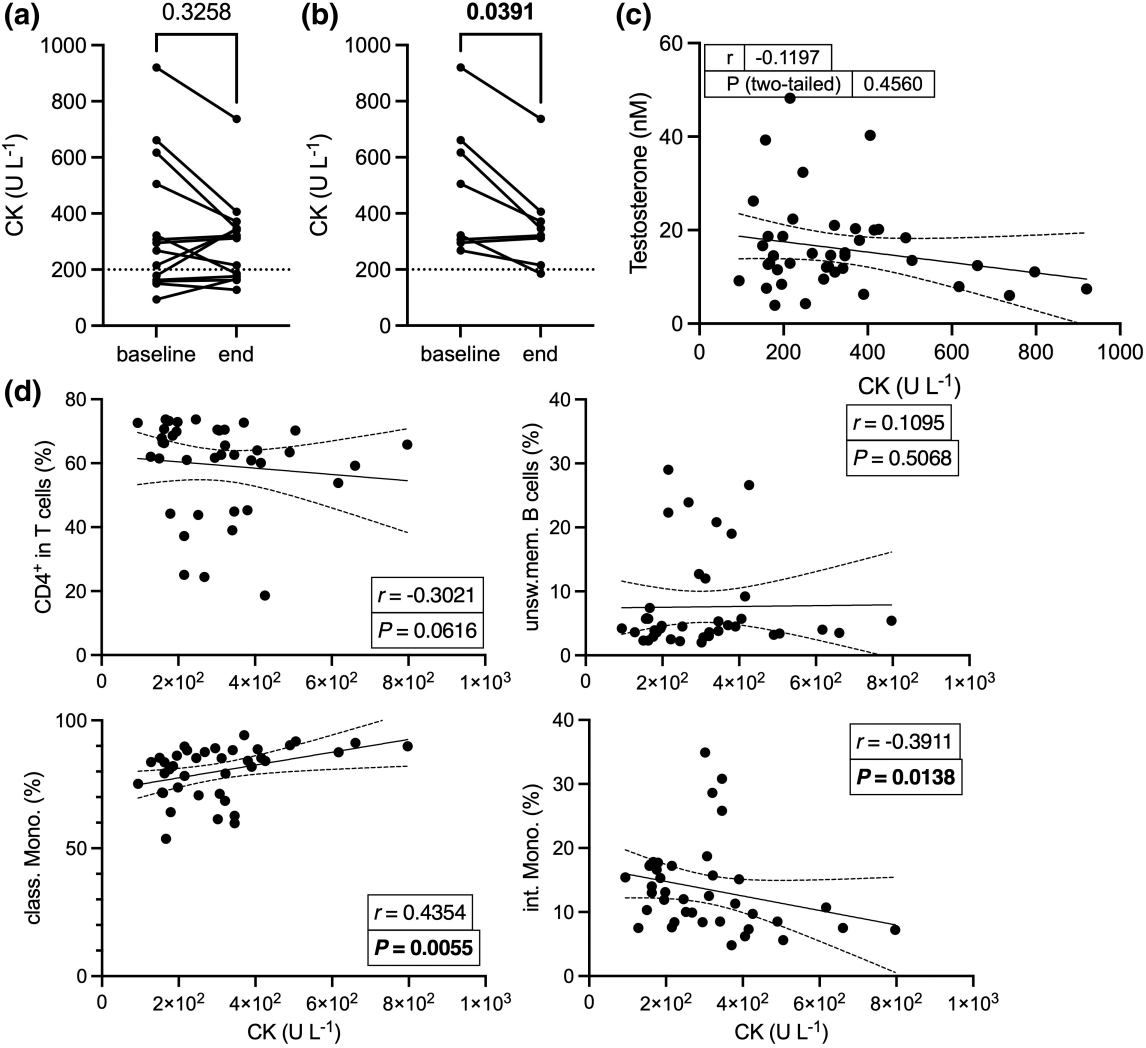
Changes in CK blood concentration and correlation with immune cell subsets. The concentration of CK in blood was measured at baseline and endpoint of the study. **(a)** shows the CK changes observed in all the participants(*n* = 14) and **(b)** in the participants with CK > 220 U L^−1^ at baseline (*n* = 8). The statistical significance of the changes was assessed using the two‐tailed Wilcoxon matched‐pairs signed rank test. The concentrations of blood CK and of testosterone **(c)**, and the frequencies of immune cell subsets **(d)** measured in samples collected at all the study time points were compiled to assess the existence of correlation between these variables using the two‐tailed Spearman's test; the correlation coefficients (*r*), and the *P*‐values are indicated.

We measured the creatine kinase (CK) in blood as a surrogate marker for muscle damage. When including all the participants into the analysis, CK concentration did not significantly change over the course of the study (*P* = 0.3258; Figure 7a). However, alternate analysis adjusted to include only the participants with high CK (above 220 U L^−1^)^33^ at baseline (8 of the 14 participants) indicated a significant CK reduction at the study endpoint (median of differences −135.5 U L^−1^, *P* = 0.0391; Figure 7b). There was no evidence of correlation between testosterone and CK concentrations (Figure 4c). Next, we assessed the existence of correlation between CK concentration and the frequency of the cell subsets that we found to be significantly altered, that is CD4^+^ T cells, unswitched memory B cells, and classical and intermediate monocytes. We identified that blood CK was correlated with the classical monocytes (Spearman's *r* = 0.4354, *P* = 0.0055), and inversely correlated with the intermediate monocyte subset (Spearman's *r* = −0.3911, *P* = 0.0138; Figure 4d). No significant relationships were found between CK and individual cytokine and chemokine concentrations (data not shown).

## Discussion

In this trial, we investigated whether 12 weeks of testosterone supplementation combined with a personalised progressive exercise training programme in men with IBM was more effective in improving the peripheral immune manifestations associated with the disease than exercise alone. Overall, testosterone supplementation was found to reduce the proportions of the classical subset of circulating monocytes and eosinophil counts. On the contrary, changes in T‐ and B‐cell subsets, and reduced concentrations of the pro‐inflammatory cytokines IL‐12, IL‐17, TNF‐α, MIP‐1β and sICAM‐1 throughout the study period occurred independently of testosterone administration and were considered to be the result of the exercise intervention. Increased frequency of the unswitched memory B‐cell subset did not translate into significant increase in anti‐C5N1A antibodies of the IgM isotype, even though such variations were observed in some of the participants.

The significance of the reduction in eosinophil counts with testosterone supplementation is uncertain as eosinophils are generally associated with allergy and atopy rather than Th1‐type inflammatory conditions such as IBM; therefore, their relevance to the disease seems unlikely. Previous research on eosinophilic inflammation in the human nasal mucosa evidenced that testosterone played an anti‐inflammatory role in these settings by negatively impacting adhesion of eosinophils to the mucosal endothelial cells and their lifespan.^34^ Similarly, the effect on eosinophil numbers induced by testosterone supplementation observed in this study may be explained by altered homing properties and/ or by a negative impact on their viability resulting in decreased numbers of these cells in peripheral blood. Of note, eosinophils have been reported to regulate DC activation, which can then influence the adaptive immune response.^35^ IL‐12p70 is a critical cytokine secreted by DC and is a critical driver of inflammatory and cytotoxic T‐cell responses.^36^ Interestingly, IL‐12p70 concentration decreased in most of the IBM participants during the course of the study. In addition, eosinophils were shown to secrete pro‐inflammatory cytokines such as TNF‐α in a chronic colitis model^37^ and also IL‐6, both mediators of acute inflammation.^38^ Consistently, we found that the prescribed exercise training was associated with a decrease in TNF‐α. Also, three of the participants had high blood concentration of IL‐6 at baseline, and at the end of the study, all of them displayed reduction in this cytokine.

We also observed changes within monocyte subpopulations after testosterone supplementation. Monocytes can be subdivided into three subsets (classical, intermediate and nonclassical) that elicit distinct functions. Classical monocytes have elevated phagocytic properties and are IL‐10 producers, whilst the intermediate subset strongly express MHC class II, a feature which plays a critical role in their specialisation to present antigens to T cells. The nonclassical monocytes are the main producers of inflammatory cytokines such as TNF‐α, IL‐1β and IL‐6.^39^ We found a significant reduction in the classical monocyte subset and a trend for MIP‐1β (macrophage inflammatory protein‐1β), a cytokine produced by macrophages and monocytes. There are two main isoforms: MIP‐1α and MIP‐1β; their role has been documented in multiple inflammatory conditions and were found to be expressed in IBM muscles.^40^ These chemokines play a role in the recruitment of monocytes, DC and T cells, thereby contributing to tissue inflammation; they also sustain the inflammatory condition by promoting the production of other cytokines including IL‐1β, IL‐6 and TNF‐α.^40^, ^41^ We detected a significantly decreased MIP‐1β concentration after the whole course of the study. We also observed a reduction of MIP‐1α in most participants, although this trend did not reach statistical significance (*P* = 0.066). Therefore, even though the size of the nonclassical monocyte subset remained not significantly affected, it is possible that their production of MIP‐1 isoforms was reduced, which was responsible for the significant reduction in TNF‐α concentrations and for the reduction trend of IL‐1β and IL‐6 that we observed.

Intercellular adhesion molecule‐1 (ICAM‐1) is a transmembrane glycoprotein constitutively expressed on endothelial cells that engage the lymphocyte function‐associated antigen (LFA‐1), an adhesion molecule expressed on T and B cells and regulate transmigration through the vascular endothelium from circulation to sites of inflammation.^42^ ICAM‐1 is also expressed on antigen presenting cells, stabilises cell interactions and promotes lymphocyte activation.^43^ sICAM‐1 is a soluble form of this adhesion molecule; sICAM‐1 competes with membrane‐bound ICAM‐1 for LFA‐1 binding, but its effect on lymphocyte behaviour remains unclear. Increased concentrations of sICAM‐1 were documented in autoimmune conditions including rheumatoid arthritis, systemic sclerosis and Graves' disease, and also in cancers.^43^ Several clinical studies used sICAM‐1 as a surrogate marker to monitor the severity of inflammation.^44^, ^45^, ^46^ Pro‐inflammatory cytokines such as TNF‐α, IL‐1 and IL‐6 are inducers of sICAM‐1.^47^ It is possible that the decrease of sICAM‐1 observed at the end of the study resulted from the combined reduction in these three cytokines.

We observed at baseline that the IBM participants displayed a high variability of testosterone concentrations and of multiple immune parameters. The changes measured following intervention were also highly variable. In order to investigate the existence of patterns, we assessed possible correlations between blood testosterone concentration and individual immunological parameters. This analysis highlighted an inverse association of testosterone with the frequency of memory CD4^+^ T‐cell subsets (effector memory and TEMRA), and with TNF‐α and MCP‐1 concentrations. However, these profiles did not translate into significantly reduced proportion of these T ‐cell subsets and of this chemokine following the testosterone supplementation arm. It is possible that exposure to increased testosterone concentration for longer than 12 weeks is required to achieve these effects.

Creatine kinase (CK) is an enzyme produced in muscles that is released upon loss of membrane integrity; its presence in blood is a sensitive marker of muscle injury. We found that the participant group with raised CK concentration at baseline demonstrated a significant reduction in this enzyme in blood at the end of the study. Our findings that CK and testosterone levels were not correlated suggest that the increase in circulating testosterone that resulted from supplementation was not directly responsible for the reduction in this biomarker for muscle damage. However, we found a positive association between CK concentration in blood and the frequency of classical monocytes, a cell subset that was significantly decreased during the testosterone administration arm of the study. This may possibly explain how testosterone indirectly impacts CK levels. The concentration of circulating pro‐inflammatory cytokines and chemokines did not correlate with CK levels, suggesting that the concentrations in blood of selected soluble immune mediators may be used as biomarkers of systemic inflammation but do not directly reflect the intensity of muscle damage. The characterisation of immune cells and cytokines within the muscles affected by IBM may possibly provide better indicators of disease severity. Muscle biopsies were an opt‐in option in our study that would have allowed us to compare immune variable changes at the systemic and at the muscle levels; however, none of the participants elected to undergo this procedure. The patients' reservations about undertaking muscle biopsies highlights the importance of identifying reliable surrogate biomarkers in blood; this provides a strong rationale for recruiting IBM patients in future studies dedicated to compare immune changes that occur in the blood and muscles.

Three separate clinical studies in elderly men with low testosterone levels (a 30‐day study with testosterone injections at onset, day 14 and 28 in 117 men,^19^ a 3‐ month study with a first injection at onset and then fortnightly in 20 men^48^ and a 30‐week study with injections at onset, week 6 and 18 in 27 men^49^) showed that testosterone administration resulted in reduced blood concentrations of TNF‐α,^19^, ^48^, ^49^ IL‐1β ^19^, ^49^ and IL‐6.^48^ Similarly to these studies, we observed a reduction of TNF‐α after testosterone supplementation. Although we observed a similar trend for IL‐1β, this change was not overall significant due to interindividual variability: IL‐1β was reduced in six participants, remained below the detection limit in six and was increased in one. Interestingly, this patient displayed no change in DHT concentration after testosterone supplementation, and his whole response to the intervention was very unique, predominantly with increased concentrations of pro‐inflammatory cytokines and chemokines. For IL‐6, although a downward trend was observed, we could not confirm the findings of these previous studies. The concentration measured at baseline for most patients did not exceed the detection threshold, and therefore, overall no significant decrease could be measured. The three patients with detectable IL‐6 at baseline all exhibited reduced concentration of this cytokine by the end of the study. Most importantly, these effects were observed after the 26‐week exercise programme, but none of these cytokines were specifically changed in a testosterone‐dependent manner. The discrepancy between our results and these previous studies may originate from various reasons: (i) the route of testosterone administration (transcutaneous cream in our study vs intramuscular^19^, ^48^ and parenteral^49^); (ii) the dose administered (100 mg daily) vs 200 mg^48^ and 1000 mg^49^; (iii) the low number of participants (*n* = 14) compared to 20,^48^ 27^49^ and 117^19^; and (iv) none of these three studies reported that exercise training was required in combination to testosterone supplementation. It is possible that in our study, the more potent effect of exercise is masking the testosterone‐dependent effect. We chose to combine testosterone supplementation on a background of exercise considering that this is the widely recommended treatment for IBM. A 2 x 2 factorial design would be ideal to understand the full effects of these two independent variables, but IBM being a relatively rare disease, it restricts the feasibility to recruit sufficient numbers of participants for this type of study.

Another aspect that may have influenced the results that we obtained is the duration of the treatment; a previous study assessing the benefit of testosterone over 36 months showed a gradual progression of muscle performance over time.^50^ Our study tested the effect of testosterone supplementation over a much shorter period of time, and therefore, the outcomes cannot be compared. It is likely that the effects of testosterone on muscle performance and immunity would be influenced by variation in pharmacokinetics between different testosterone formulations, and by different durations of treatment, as well as to remaining muscle strength and mass which may be nonimmune related.

### Limitations

Whilst there are a number of strengths associated with the project, including the detailed cellular and molecular immune‐phenotyping, there are also a number of limitations which should be noted. First, the size of the cohort enrolled in this study was small (14 participants). As IBM is a relatively rare condition, our study involved only male patients recruited from a single centre and we could not enrol as many participants as would have been necessary to achieve optimal analytical power, which possibly generated type II errors. Second, we found that the increase in circulating testosterone concentrations that resulted from supplementation was inversely correlated with disease duration. To our knowledge, there is no solid biological basis for this observation. However, one possibility is that the men with more advanced disease and more severe loss of manual strength and dexterity found it more difficult to apply the cream and to facilitate optimal testosterone uptake. Third, the stage of disease progression was quite variable within our group of participants, with some patients walking unassisted whilst others relied on a wheelchair for mobility; taking these differences into consideration, each participant's exercise programme was personalised to suit their physical abilities, and as a result, varied considerably in intensity. The cross‐over design of our study was intended to alleviate these biases; however, it would be of utmost interest to repeat this study with larger groups of patients at similar stages of the disease; this will allow a more consistent exercise programme, and presumably will generate more consistent outcome measures. Fourth, we tested simultaneously 14 different pro‐inflammatory cytokines and chemokines in serum samples and although the method used was very sensitive, several of these molecules were below detection levels and variations could not be identified. It is likely that their production by immune cells infiltrating the affected muscles result in much higher concentrations locally than we detected in peripheral blood. It would be interesting to investigate in muscle biopsies whether similar changes in these pro‐inflammatory molecules could be observed. Likewise, investigating the modifications affecting the immune cells recruited and reactive within the muscles would be critical to confirm that the information obtained from the blood provides a valid biomarker of muscle inflammation. Lastly, in the absence of a control group in which participants would have received placebo without a personalised exercise background, we cannot exclude the possibility that some of the results that have been attributed to exercise might actually have resulted from a placebo or a non‐specific response. However, we consider exercise to be a standard of care, and therefore regard a study to not include exercise unethical.

## Conclusions

Our study showed that men with IBM undergoing an individualised regular exercise training routine over a period of 26 weeks, exhibited reduction in a number of immune mediators of inflammation and that 12 weeks of testosterone supplementation had some additional measurable effects on monocyte subpopulations and cytokines. The immunoregulatory effects were associated with reduced CK levels in six of the eight participants who experienced raised blood concentration at baseline of this muscle injury marker. This work further emphasises that maintaining a regular level of physical exercise helps in controlling inflammation in IBM, and therefore possibly, the speed of progression of the disease. Based on these findings and the response to intervention observed in some of the participants, we propose that incorporating testosterone supplementation with a programme of exercise training may have some synergistic anti‐inflammatory effects in patients with IBM. However, the optimal nature and duration of the intervention and the stage of disease progression when the intervention provides most potent anti‐inflammatory effects will need to be further characterised and refined to achieve optimal outcomes.

## Methods

### Study design

The protocol used in this study has been detailed by us elsewhere.^31^ In brief, a cross‐over design was employed, with participants randomised 1:1 to either placebo or testosterone arms for an initial 12‐week treatment period. Following a 2‐week washout period, participants then ‘crossed‐over’ to receive the alternate treatment for the second 12‐week treatment period. The trial was conducted as a double‐blinded trial, and participants completed the prescribed exercise programme across the entire 26‐week study period. The study drug (testosterone or placebo) was self‐administered daily via a transdermal cream.

### Participants

Fourteen male IBM patients were successfully enrolled to the study, recruited from the Myositis Clinic at the Centre for Molecular Medicine and Innovative Therapeutics (CMMIT). All participants had been diagnosed with IBM by a neurologist. Seven of the participants enrolled had clinicopathologically defined IBM, three had clinically defined IBM according to the ENMC criteria^51^ and four had clinically confirmed diagnoses on clinic‐serological grounds with consistent neurophysiology, but no biopsy available. The eligibility criteria have been fully detailed in Connor *et al.* ^31^; to summarise, inclusion criteria were as follows: male adults with a diagnosis of IBM able to provide informed consent, serum testosterone <14 nmol L^−1^, normal haematocrit and prostate‐specific antigen (PSA) concentration, no clinical suspicion of prostate malignancy; whilst the exclusion criteria were pituitary or testicular disease contributing to androgen deficiency, cardiovascular disease (unstable angina, myocardial infarction, stroke, transient ischaemic attack or cardiac revascularisation procedure within the previous 12 months), history of prostate cancer, major medical comorbidity other than cardiac pathology, treatment with anti‐androgen drugs for benign prostatic hyperplasia; elevated blood pressure (> 160/100 mm Hg); abnormal prostate examinations suggesting malignancy; total cholesterol > 6.5 mmol L^−1^ or LDL > 4.0 mmol L^−1^; eGFR < 45 mLmin^−1^. None of the participants had received immunosuppressive treatments recently, or during the course of the trial.

### Treatment: testosterone and placebo creams

Testosterone was provided as a transdermal cream (AndroForte5™, 50 mg mL^−1^); 100 mg of testosterone (2 mL) was applied by the participant to the same area of the torso with low subcutaneous fat daily. The placebo cream contained identical inactive ingredients to the treatment cream and was indistinguishable by smell or appearance. Both creams were supplied by Lawley Pharmaceuticals, WA, Australia.

### Exercise programme

The prescribed exercise programme, to be performed at least three times a week, composed of a combination of light‐to‐moderate resistance training, balance training and aerobic exercises for periods of 30–60 min repeated at least three times per week, and was adjusted according to the extent of impairment of individual patients' motor functions, as detailed in Connor *et al*.^31^

### Compliance with study interventions

The participants were provided with compliance diaries, in which they had to record periods of exercise training and cream application. Also, the participants were requested to return unfinished tubes of cream at the end of each study arm, which allowed the investigator to control that the remaining quantity reflected the expected cream usage.

### Sample collection

Peripheral blood samples were collected by venepuncture at the following time points: Baseline (commencement of the study treatment), Week 12 (end of the first study arm) and Week 26 (end of the second arm and study). For flow cytometry analysis of circulating leukocytes, blood samples were collected by venepuncture in BD Vacutainer® Lithium‐heparin spray‐coated tubes (BD Biosciences, Macquarie Park, NSW, Australia). For serum analysis, blood was collected in BD Vacutainer® SST™ (BD Biosciences).

### Measurement of circulating testosterone and DHT

Testosterone is converted into 5α‐dihydrotestosterone (DHT) though the enzymatic activity of 5‐alpha reductase. DHT binds to the androgen receptor with greater affinity than testosterone, resulting in a more potent androgenic activity.^52^ We measured DHT as an added marker of treatment efficacy. Serum testosterone and DHT were determined by an in‐house developed liquid chromatography–tandem mass spectrometry (LC–MS/MS) assay performed on a Waters Xevo TQ‐S tandem mass spectrometer coupled with a Waters ACQUITY® H‐Class UPLC system with quaternary pumping capability (Waters Corporation, Milford, MA, USA). Experimental set‐up was programmed by Waters MassLynx software. The steroids were ionised by electrospray ionisation and detected in the positive‐ion mode. Data processing and quantitation were performed by the TargetLynx Application Manager. Calibration was performed using a 7‐point curve through linear regression with fit weighting to 1/x. This assay was validated according to the guidelines set out by Clinical and Laboratory Standards Institute (CLSI C57‐ED1).^53^ Assay recovery, linearity, carryover, lower limits of detection, lower and upper limits of quantitation, accuracy, intraday and interday precision, sample stability were assessed against the criteria outlined by the aforementioned guidelines.

### Measurement of creatine kinase

Creatine kinase (CK) was measured in blood by Pathwest laboratory medicine WA according to a routine diagnostic laboratory methodology, using the Abbot Architect reagents and Platform (Abbot Laboratories, Abbot Park, IL, USA) and following the manufacturer's recommendations.

### Analysis of immune cell populations in the blood by flow cytometry

The main leukocyte populations and subsets were analysed by flow cytometry. Freshly collected blood samples were stained using several panels of antibodies. The fluorochrome‐conjugated antibodies used for cell staining were as follow: anti‐ CD3 (UCHT1) BV510, CD4 (OKT4) FITC, CD8 (SK1) APC‐H7, CD11c (B‐Ly6) PE‐Cy7, CD19 (HIB19) FITC, CD27 (M‐T271) BV421, CD56 (B159) PE‐CF594, CD61 (VI‐PL2) FITC, CCR7 (3D12) PE, IgD (IA6‐2) FITC, HLA‐DR (G46‐6) BV421 (all purchased from BD Biosciences), and CD3 (UCHT1) APC FIRE 750, CD14 (63D3) Alexa Fluor 700, CD16 (3G8) Alexa‐Fluor 647, CD19 (HIB19) APC FIRE 750, CD45 (HI30) BV510, CD45RA (HI100) APC, CD123 (6H6) PE, TCRαβ (IP26) PE‐Cy7 (all purchased from BioLegend, Wangara, WA, Australia). Antibody panel mixes were diluted in BD Horizon™ brilliant stain buffer (BD Biosciences), added onto the blood samples for 30 min at room temperature for staining. The erythrocytes cell fraction was removed using BD FACS™ lysing solution (BD Biosciences) for 10 min at room temperature as per the manufacturer's recommendations. After two consecutive centrifugations (300 *g* for 10 min each), each sample was resuspended in PBS supplemented with 2% foetal calf serum. For cell count normalisation, a defined number of flow‐Count Fluorospheres (Beckman Coulter, Lane Cove, NSW, Australia) was added to each sample prior to acquisition on a Gallios™ flow cytometer (Beckman Coulter). We have optimised our cell labelling protocol to minimise the number of steps to prevent cell alteration and loss, so that to obtain an accurate snapshot of the immune cell populations. The flow cytometry data were analysed using FlowJo™ software (Beckton Dickinson, Ashland, OR, USA). The gating strategy that we used for stratifying the cell populations into subsets is illustrated in Supplementary figure 1 for monocytes and NK cells and in Supplementary figure 2 for T and B cells.

### Measure of chemokines and cytokines in serum

For serum analysis, the serum fraction was separated from fresh blood by centrifugation at 1300 *g* for 15 min, divided into small aliquots and stored at −80°C until analysis to avoid protein degradation otherwise due to freeze/thaw cycling. The serum aliquots were thawed on ice just prior to analysis in duplicate using ProcartaPlex™ 14‐plex immunoassay (ThermoFisher Scientific, Scoresby, VIC, Australia) according to the manufacturer's recommendations, and the following molecules were tested (the lower and upper limits of quantification [LLOQ and ULOQ] are indicated in pg mL^−1^): IP‐10 (2–2075), MCP‐1 (3.9–16 100), MIP‐1α (2–2088), MIP‐1β (5.6–5775), sICAM‐1 (142–582 000), GM‐CSF (15–59 600), IFN‐α (0.6–2550), IFN‐γ (15–61 900), IL‐1α (0.7–2850), IL‐1β (2–8150), IL‐6 (10–42 900), IL‐12p70 (6.2–25 200), IL‐17A (2.3–9300), TNF‐α (8.6–35 200). The washing steps were performed using a Bio‐Plex Pro™ II Wash Station (Bio‐Rad, Gladesville, NSW, Australia) and detection using a Luminex MAGPIX® system (Luminex Corporation, Austin, TX, USA); the data generated were analysed using the Luminex xPONENT® software (Luminex Corporation). Where a molecule was measured at a concentration outside of the quantification range, the corresponding LLOQ or ULOQ value was used as a substitute.

### Anti‐NT5C1A antibodies ELISA

Anti‐NT5C1A antibodies were analysed in the serum using an in‐house semi‐quantitative ELISA methodology adapted from Bundell *et al*.^54^ Flat bottom microtitre 96‐well plates (Maxisorp, Nunc, Roskilde, Denmark) were coated with 10 μg mL^−1^ of NT5C1A protein (GenScript, NJ, SA) diluted in 50 mMm carbonate–bicarbonate buffer pH 9.6 for 2 h at ambient temperature. Wells were washed with PBS/0.1% Tween (PBST) and saturated with blocking buffer (PBST / 5% skim milk powder) overnight at 4°C. After washing with PBST, 100 μL of patient serum diluted to 1:1000 in blocking buffer was added in duplicate and incubated for 2 h at ambient temperature for anti‐NT5C1A antibody capture. Then, wells were washed with PBST before adding horseradish peroxidase (HRP)‐conjugated anti‐human secondary antibodies directed against pan IgG/M/A or IgG or IgM or IgA (Invitrogen, Rockford, IL, USA) and incubating for 1 h at ambient temperature. Wells were washed as above before revelation by incubation in 50 μL TMB solution (Thermo Fisher Scientific, Waltham, MA, USA) for 10 min before stopping the colorimetric reaction with 50 μL of 2 M H2SO4 solution. The absorbance at 450 nm was read using a microplate reader FLUOstar Omega (BMG Labtech, Mornington, VIC, Australia). Each plate included a positive control made of anti‐NT5C1A antibodies purified from the serum of a previously identified seropositive patient diluted in blocking buffer at 2 μg mL^−1^. Pooled serum from healthy controls diluted 1:1000 in blocking buffer was used as a negative control. Blank duplicates were obtained by performing all the steps in the absence of a sample. Absorbance values obtained for patients' samples were recorded as a fold change relative to the healthy serum pool minus the averaged duplicate blank values. Reference cut‐off values for seropositivity correspond to the 99^th^ percentile of the value obtained with pooled healthy sera (Busselton Population Health Study participants, *n* = 190).

### Statistical analysis

The different variables were measured at the end and start of the study periods, and the statistical significance of the changes tested with the nonparametric Wilcoxon matched‐pairs signed rank test. In order to account for multiple comparisons for multiplex cytokine and chemokine measure analysis, the *P*‐values were adjusted using the Holm–Bonferroni's correction method. For analysis of unpaired data sets, the nonparametric Mann–Whitney *U*‐test was used. In intervariable correlation analysis, we compiled the testosterone concentrations measured for all participants at all time points that we associated with the indicated immunological variables measured in the corresponding samples; Spearman rank correlation coefficients (*r*) and *P*‐values were calculated. These statistical tests to compare differences or association were computed using the GraphPad Prism® v9.3.1 (San Diego, CA, USA) statistical analysis package, and differences were considered significant when *P* < 0.05 (two‐tailed).

## AUTHOR CONTRIBUTIONS

**Jerome D Coudert:** Conceptualization; data curation; formal analysis; investigation; project administration; supervision; writing – original draft; writing – review and editing. **Nataliya Slater:** Formal analysis; investigation. **Anuradha Sooda:** Formal analysis; investigation. **Kelly Beer:** Data curation; investigation; project administration. **Ee Mun Lim:** Investigation; methodology. **Conchita Boyder:** Methodology. **Rui Zhang:** Methodology. **Frank L Mastaglia:** Writing – review and editing. **Yvonne C Learmonth:** Conceptualization; formal analysis; investigation; writing – review and editing. **Timothy J Fairchild:** Conceptualization; investigation; writing – review and editing. **Bu B Yeap:** Conceptualization; investigation; writing – review and editing. **Merrilee Needham:** Conceptualization; funding acquisition; investigation; writing – review and editing.

## Conflict of Interest

Active medication and placebo were provided without cost by Lawley Pharmaceuticals, Western Australia. BBY has received speaker honoraria and conference support from Bayer, Lilly and Besins, research support from Bayer, Lilly and Lawley Pharmaceuticals, and held advisory roles for Lilly, Besins, Ferring and Lawley Pharmaceuticals. The other authors have no competing interests to declare.

## Ethics approval

This study was approved by the Human Research Ethics Committee of Murdoch University (#2017/262).

## Supporting information


Supplementary material
