# Supplementary material for: Immunoregulatory effects of testosterone supplementation combined with exercise training in men with Inclusion Body Myositis: a double‐blind, placebo‐controlled, cross‐over trial
Source: Clin Transl Immunology. 2022 Sep 22;11(9):e1416. doi: 10.1002/cti2.1416 (PMC9495304; doi:10.1002/cti2.1416)
Supplement: Supplementary file 1 — Supplementary material [file CTI2-11-e1416-s001.pdf]

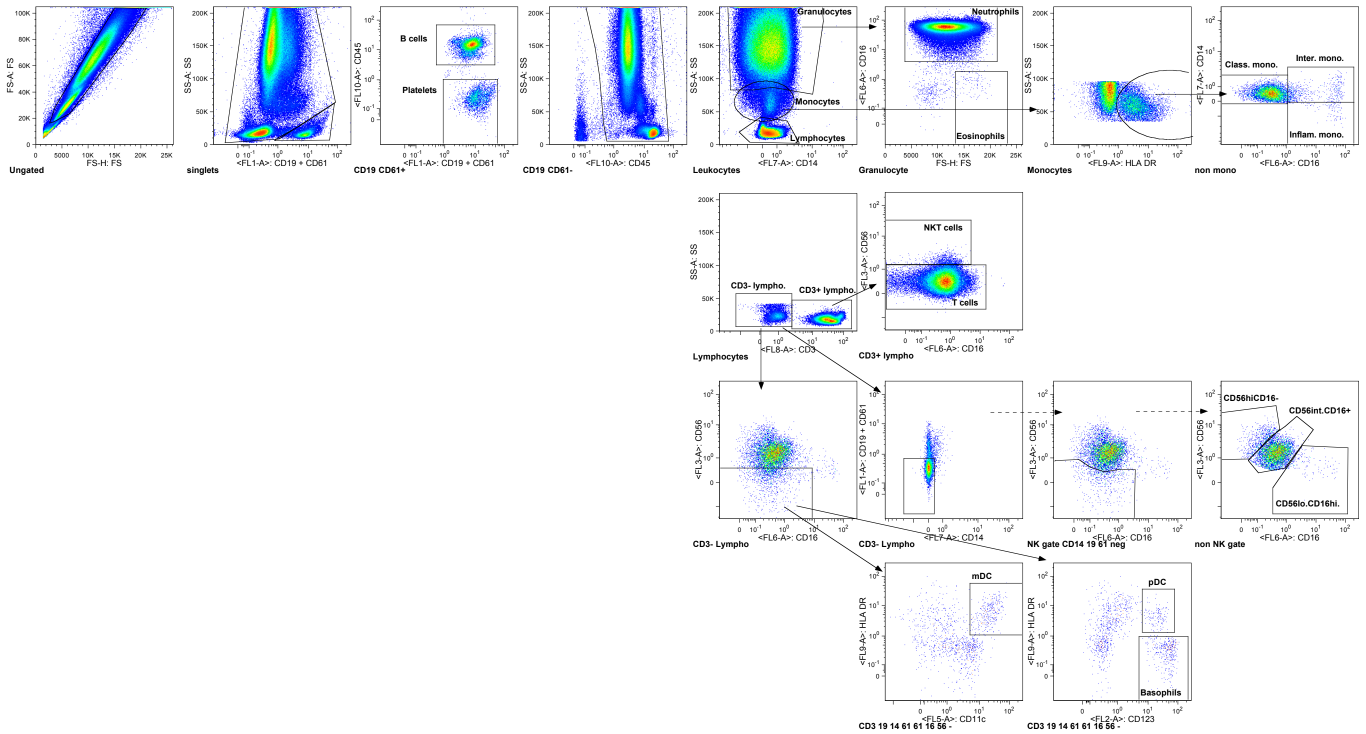

Supplementary figure 1:
Gating strategy of flow cytometry data for analysis of leukocyte populations and subsets of monocytes and NK cells. The surface markers used for gating at each step of the analysis are indicated on the x and y axis. The name of the identified leukocyte populations are indicated next to their respective gating areas within the flow cytometry dot plot panels.

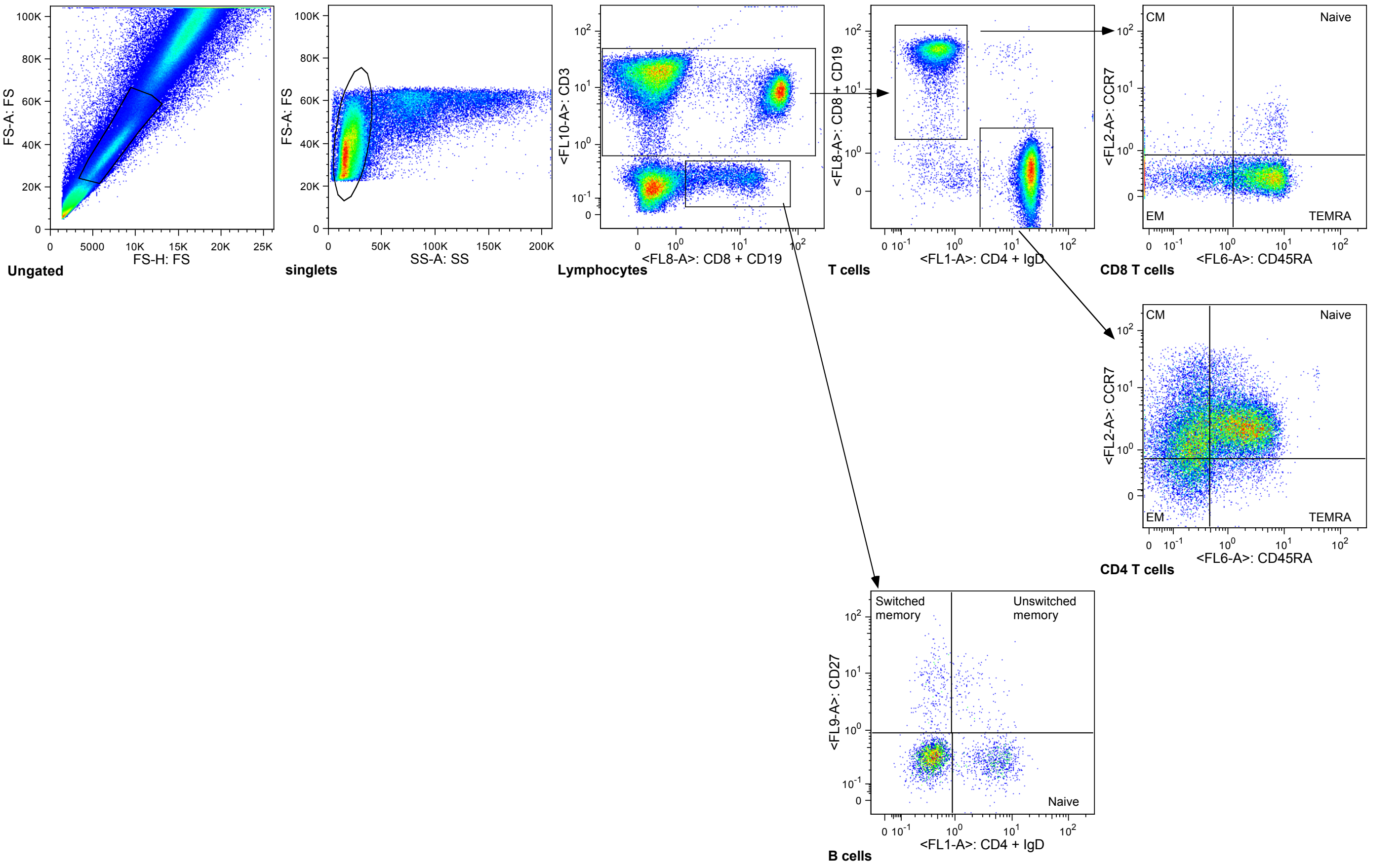

Supplementary figure 2:
Gating strategy of flow cytometry data for analysis of T and B cell subsets. The surface markers used at each step of the analysis for T and B cell subset gating are indicated on the x and y axis. The names of the gated cell subsets are indicated below their respective dot plot panels and the stage of differentiation within their respective quadrants.

		Baseline	1st arm		Wash-out	2nd arm	
ID	year at IBM onset	age	Treatment	Deviation	Deviation	Treatment	Deviation
1002	2003	78	P	0	0	T	0
1003	1995	78	P	0	0	T	-7
1004	2014	48	T	+7	+7	P	+11
1005	2015	67	P	0	0	T	-1
1006	2014	69	T	0	+1	P	-1
1007	1995	71	P	0	0	T	0
1008	2009	81	T	0	+11	P	+1
1009	2006	66	P	0	+1	T	-1
1010	2000	61	T	0	0	P	0
1011	2007	66	T	0	0	P	0
1012	2003	45	P	0	0	T	0
1013	2013	57	P	0	0	T	0
1014	2012	74	T	0	0	P	0
1015	2007	79	T	0	0	P	0

Supplementary Table 1:
Participant demographics, treatment timing and deviation to protocol. The number of years since initial IBM symptoms (onset), and the age of the participants at baseline are indicated. The treatments received during each arm of the study are reported as P= placebo and T= testosterone. The deviation time in days relative to the planned 12-week duration for each arm of the study and 2-week duration of the wash-out period are indicated.

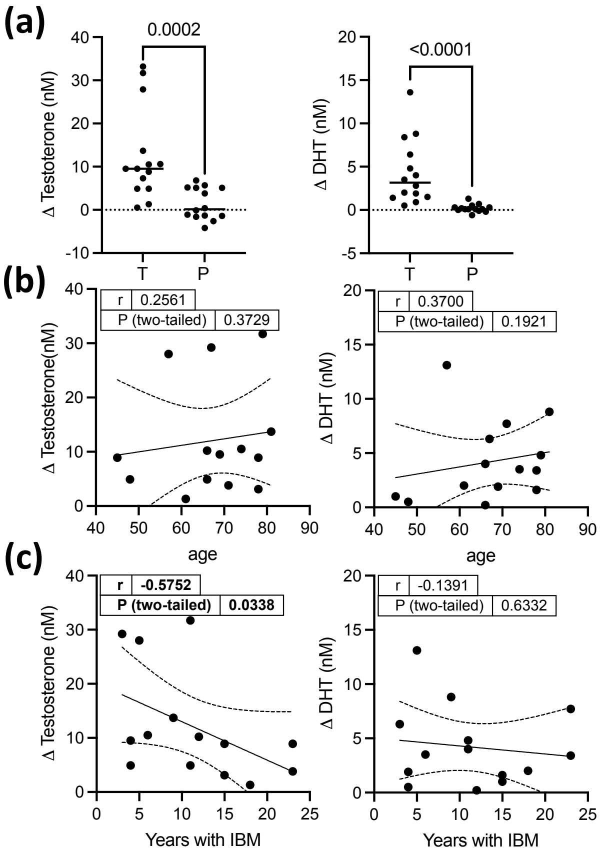

Supplementary Figure 3:
Changes of Testosterone and DHT concentrations during study arms, and correlation with patients' age and disease duration. (a): Changes of testosterone and DHT concentrations in serum measured between baseline and the end of each testosterone (T) or placebo (P) study arm. The significance of the concentration changes were assessed using the two-tailed Mann-Whitney test; P-values are indicated. Correlation analysis between the variation of testosterone (left panel) or of DHT concentrations (right panel) and the participants' age (b), or the number of years since disease onset (c). The correlation coefficients (r) and P-values were calculated using the two-tailed Spearman's test; where significant (< 0.05) the P-value was highlighted in bold).

	Median of differences (baseline-onset)	P value (Wilcoxon matched-pairs signed rank test)	Median at baseline of study	Median at end of study
<u>**Leukocyte populations (absolute numbers / L of blood)**</u>				
**T cells**	-1,563,500	0.6698	843,313,500	669,567,500
**NKT cells**	-1,547,302	0.4263	10,583,946	10,121,743
**NK cells**	-8,958,827	0.1937	116,847,700	101,867,440
**B cells**	-5,669,500	0.6698	136,972,500	147,943,000
**Monocytes**	-15,882,000	0.7609	115,907,000	90,587,000
**mDC**	-5,293,673	0.3258	15,237,120	15,753,800
**pDC**	-1,138,009	0.1726	3,627,162	1,810,223
**Neutrophils**	-432,018,000	0.7148	2,427,852,500	2,180,349,000
**Basophils**	-2,228,725	0.1189	8,495,084	8,226,162
**Eosinophils**	-4,540,501	**0.0203**	7,916,152	5,245,493
<u>**Leukocyte subsets (% within subset)**</u>				
**Monocytes**				
classical	-2.75	**0.0494**	84.75	80.1
intermediate	3	**0.0419**	10.5	15.95
non-classical	-1.2	0.7148	3.6	4.15
**CD4+ T cells**				
naïve	-1.5	0.2958	43.7	43.5
CM	1.15	0.1189	41.1	43.35
EM	0.75	0.5950	11.3	11
TEMRA	-0.2	0.0625	2.2	1.6
**CD8+ T cells**				
naïve	0.8	**0.0083**	7.7	8.3
CM	0.1	0.9384	3.4	3.7
EM	0	0.3311	42.5	46
TEMRA	-2	0.0867	37	31.5
**B cells**				
naïve	1	0.2991	48	47
unswitched mem.	0.95	**0.0491**	3.95	5.5
switched mem.	0.35	0.5309	7.6	6.6
IgD,CD27 DN	-2.5	0.1724	34	31.5

Supplementary Table 2:
Size of immune cell populations and subset proportions at baseline and end-point of study. The median changes were measured between baseline and end-point collection time points for the cell counts of leukocyte populations and for the proportion of the indicated cell subsets. The statistical significance of the changes was assessed using the Wilcoxon matched-paired signed rank test; where significant (P-value < 0.05), the values are highlighted in bold. The median values at baseline and end of study of the leukocyte population cell counts and cell subset proportions are also indicated.

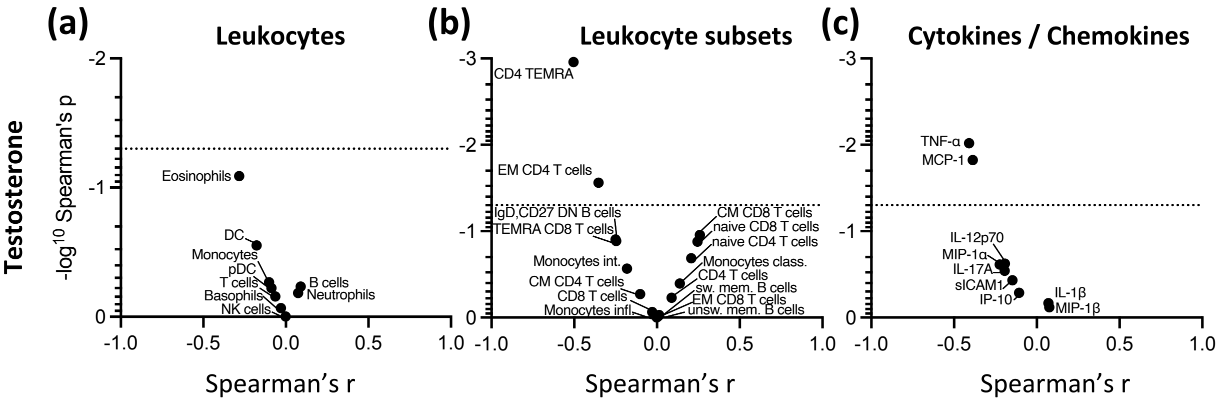

Supplementary Figure 4:
Correlation of testosterone concentration with immune cells and soluble immune mediators in blood. The testosterone concentration measured in each sample collected during the study was assessed for correlation with the indicated immune variables measured in the corresponding sample using the two-tailed Spearman-ranked test. The calculated Spearman coefficients r and the P-values were graphed as -log10 Spearman's P values (x axis) against Spearman's r values (y-axis) for each data set: leukocyte population counts (a), proportions of leukocyte subsets (b) and concentrations of cytokines and chemokines (c). The dashed lines indicate the significance threshold (P = 0.05); the correlation of testosterone with the variables situated above this line were considered significant (negative correlation when -1 < r < 0; positive correlation when 0 < r < +1).

Coudert et al.
Supplementary Figure 4

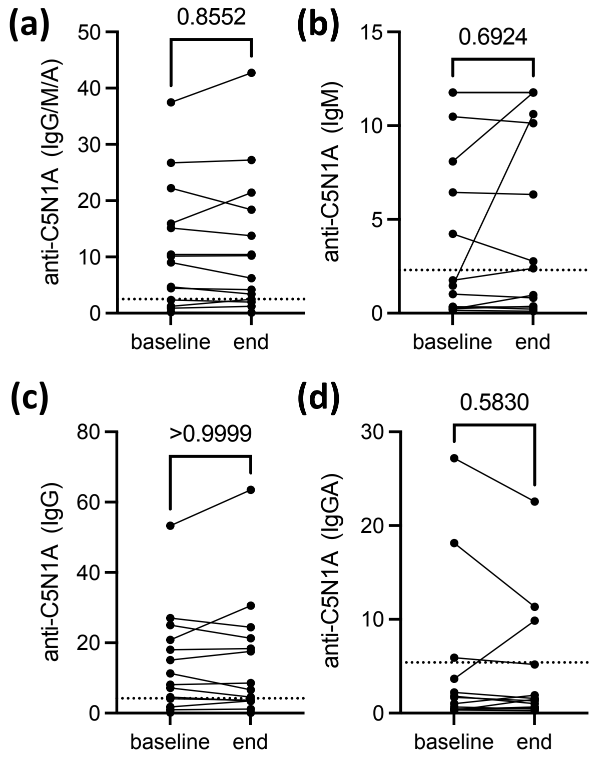

Supplementary Figure 5:
Variation of anti-C5N1A autoantibody concentration during study. Autoantibodies directed against C5N1A were measured in blood samples collected at study baseline and end point. The concentration of total anti-C5N1A antibodies (IgG/M/A) (a), and of those of the IgM (b), IgG (c) and IgA (d) isotype were measured by ELISA. The dotted lines represent the cut-off values for seropositivity. The statistical significance of the concentration changes between the 2 collection time points were assessed using the two-tailed Wilcoxon matched-pairs signed rank test; the calculated P-values are indicated.
